# Rosai–Dorfman Disease (Sinus Histiocytosis with Massive Lymphadenopathy) of the Pancreas: Third Reported Occurrence

**DOI:** 10.1007/s12029-012-9424-z

**Published:** 2012-08-04

**Authors:** Minerva A. Romero Arenas, Aatur D. Singhi, Ralph H. Hruban, Andrew M. Cameron

**Affiliations:** 1 Sinai Hospital of Baltimore, 2401 W. Belvedere Ave., Baltimore, MD 21204 USA; 2 Department of Pathology, University of Pittsburgh Medical Center Presbyterian A616.2, 200 Lothrop Street, Pittsburgh, PA 15213-2546 USA; 3 The Sol Goldman Pancreatic Cancer Research Center, Department of Pathology, Johns Hopkins University School of Medicine, Weinberg 2242, 401 Broadway, Baltimore, MD 21231 USA; 4 The Johns Hopkins Hospital, Ross 765, 720 Rutland Ave, Baltimore, MD 21205 USA

## Introduction

Sinus histiocytosis with massive lymphadenopathy (SHML) was first described in 1969 by Rosai and Dorfman [1]. It is a rare inflammatory disorder with key clinicopathological characteristics such as *emperipolesis* and positive immunostaining for S-100 protein [2] and CD68. Rosai–Dorfman disease (RDD) is primarily manifested in the lymph nodes, yet extranodal disease has been reported in as many as half of patients [3]. Such cases affect primarily the head and neck, upper respiratory tract, and central nervous system, although the disease has been found in almost every organ system. The gastrointestinal tract is rarely involved, and only two cases of primary involvement of the pancreas have been previously described [4, 5].

## Case Presentation

**Fig. 1:**
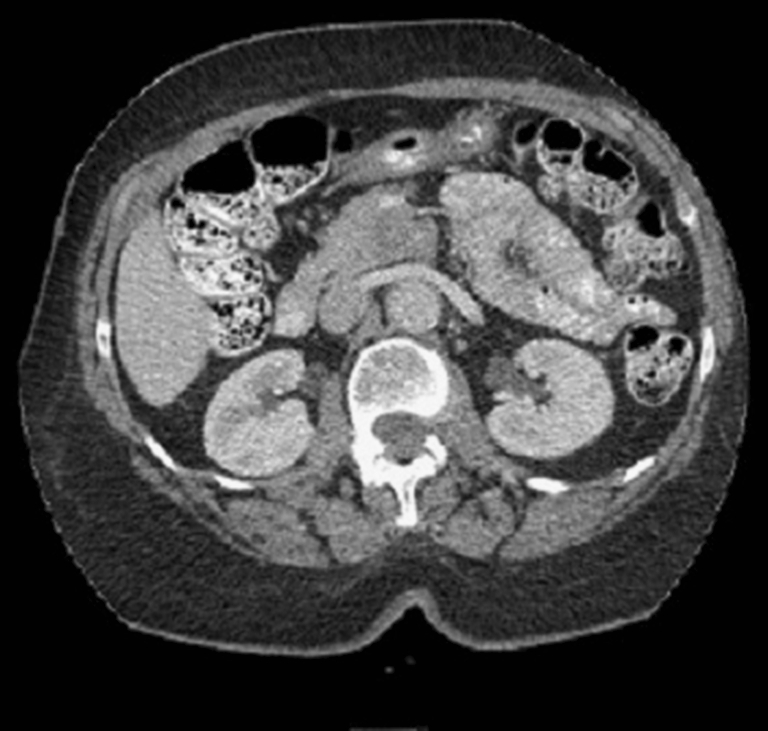
Computed tomography demonstrating a subtle mass in the head of the pancreas, posterior to the portal confluence

We present a 74-year-old African–American female who presented to another hospital with low-grade periumbilical pain which she described as “bloating,” but she had experienced no weight loss. Computed tomography of the abdomen demonstrated a mass in the head of the pancreas. There was no radiographic evidence (See Fig. 1) of lymphadenopathy or obvious metastatic disease. The mass was located in the uncinate process and appeared closely adherent to the superior mesenteric vein (SMV) and superior mesenteric artery (SMA). The patient denied nausea or vomiting and was not jaundiced, and her CA 19-9 was within normal limits. The patient was referred for surgical evaluation. After review of her imaging and clinical presentation, it was determined that this mass was suspicious for malignancy. After discussing options with the patient, she opted for surgical intervention. The patient underwent a pylorus-sparing pancreaticoduodenectomy after preoperative medical evaluation and optimization. Intraoperatively, there was no evidence of distant spread, and the mass appeared locally resectable. However, the portal vein was intimately adherent to the specimen and was resected due to friability and bleeding. The portal vein was reconstructed in our standard fashion with primary anastamosis, and the rest of the resection and reconstruction were uneventful.

## Pathologic Findings

The specimen contained a poorly circumscribed mass centered within the uncinate process of the pancreas and abutting the vascular groove. It was firm and, on cut section, consisted of yellow–white homogeneous parenchyma. The greatest dimension measured 2 cm in diameter. The lesion grossly appeared to infiltrate the wall of the adherent SMV. The remaining pancreas was grossly unremarkable without evidence of additional lesions or disease.

### Microscopic Findings

**Fig. 2:**
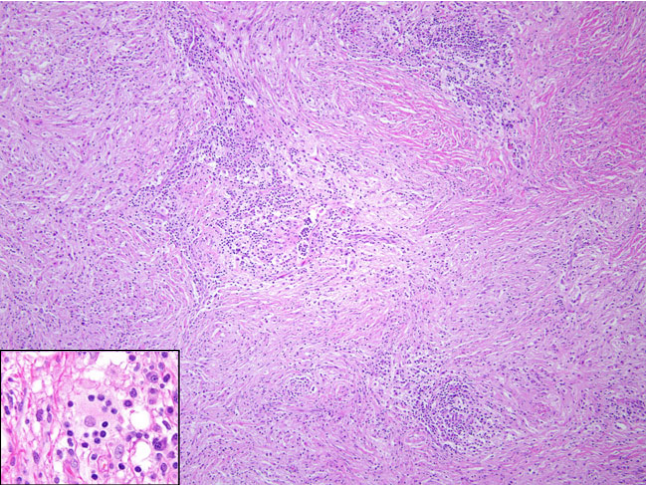
Hematoxylin and eosin-stained section demonstrating two distinct cell populations of polygonal- to spindled-shaped histiocytes (*pale areas*) and scattered lymphoid aggregates (*dark areas*). *Inset*: An area of lymphocyte engulfment by a lesional histiocyte consistent with emperipolesis

**Fig. 3:**
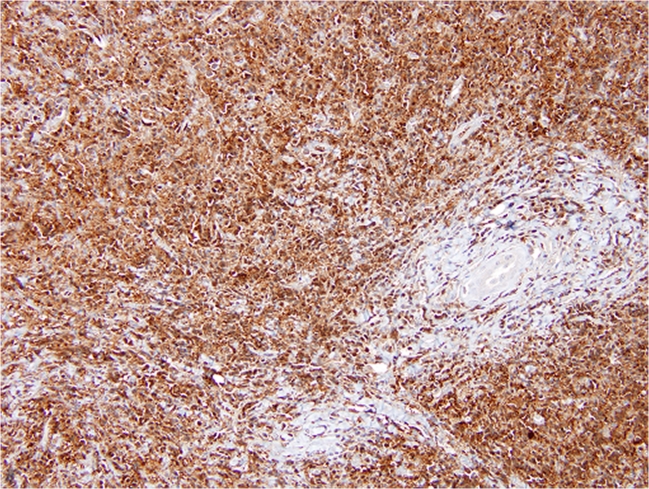
Immunohistochemical labeling for S-100 protein was diffusely positive within the lesional histiocytes

At low magnification, the mass was infiltrative and composed of a polygonal-to-spindle cell population of histiocytes (pale areas) and scattered small- to medium-sized lymphoid aggregates (darker areas). The histiocytes demonstrated an insidious invasive pattern as they subtly penetrated and entrapped the surrounding normal pancreatic elements and extended through the media of the SMA. The histiocytes had abundant, granular eosinophilic cytoplasm with a round-to-oval nucleus. Emperipolesis was conspicuous and readily identified, particularly within lymphatic-like spaces (Fig. 2, inset). Immunohistochemical labeling for CD-68 and S-100 protein (Fig. 3) was diffusely positive within the histiocytic population confirming the diagnosis of extranodal Rosai–Dorfman disease. Immune stain for IgG4 was noncontributory due to high background. Twelve lymph nodes were also benign.

## Discussion

Rosai and Dorfman first described a clinicopathological entity separate from histiocytosis X in 1969 [1] and named it SHML. Rosai–Dorfman disease is characterized by emperipolesis or lymphophagocytosis, where affected nodes show expansion of the sinuses by histiocytes with an abundant pale eosinophilic cytoplasm often containing lymphocytes within. All cases involving the gastrointestinal tract have strongly expressed CD68 and S-100 protein [2]. Classically, patients present with painless lymphadenopathy, which is frequently self-limited in course. The disease presents in children and young adults though it can affect all ages. There is a slight predilection for male patients (58 %) and African–American ethnicity [3]. Its etiology is yet to be determined, though current thinking attributes it to an immunologic process.

Occasionally, patients have had an indolent course where the disease has spread retrograde from the lymph nodes into other organ systems. Notably, one case report described the 17-year-long course of a patient who presented at 11 years of age with nasal disease and ultimately died of diffuse involvement of his lungs, kidneys, and pancreas [6]. Although, the disease was originally defined as a nodal disorder, extranodal involvement has been reported in as many as half of all patients [3].

Multiple organ systems have been involved in extranodal disease, notably the head and neck, eyes and orbits, and the central nervous system. The gastrointestinal system is rarely involved, and only two cases of primary pancreatic involvement have been reported. Esquivel first reported a 48-year-old African–American female with a 3.5-cm mass in the body of the pancreas, who underwent a distal pancreatectomy. This patient did not have lymphadenopathy, and preoperative biopsies had been inconclusive [5]. Zivin reported a second case in 2009, a 63-year-old African–American female with obstructive jaundice and weight loss, who was found to have a mass in the head of the pancreas. Preoperative fine-needle aspiration was negative for malignancy [5].

It is important to distinguish patients with RDD from those with autoimmune pancreatitis (AP). In addition to chronic pancreatitis, the characteristic histologic features of AP are dense lymphoplasmacytic infiltrate centered on the pancreatic ducts, venulitis, and interstitial fibrosis. Increased numbers of IgG4+ plasma cells are also seen in most cases of AP [7]. The pathologic findings in our patient were classic for RDD, and the IgG4 stain was noncontributory due to high background. Furthermore, IgG4+ cells have been documented in sclerosing diseases, such as AP, but the association between IgG4+ plasma cells and RDD is not yet well understood. Two studies evaluating this characteristic have been reported. In extranodal (pulmonary) RDD, 10–70 % of plasma cells were IgG4+ [7]. In patients with cutaneous RDD, the mean number of IgG4+ plasma cells was 349 cells/HPF [8]. This area needs further study in larger patient populations before conclusions may be drawn in individual cases.

Patients who present with a pancreas mass, abdominal discomfort, weight loss, and malaise present a challenge to clinicians evaluating them for malignant process as this rare entity may present in an indistinguishable manner. In patients whose biopsy results are inconclusive or negative for malignancy despite a clinical picture of neoplasm, performing immunohistochemical labeling may be a way to discard RDD from the differential although the vast majority of patients presenting in such a fashion will have their diagnosis made upon resection.
